# Effects on Vaginal Microbiota Restoration and Cervical Epithelialization in Positive HPV Patients Undergoing Vaginal Treatment with Carboxy-Methyl-Beta-Glucan

**DOI:** 10.1155/2020/5476389

**Published:** 2020-04-27

**Authors:** Giada Lavitola, Luigi Della Corte, Nicoletta De Rosa, Carmine Nappi, Giuseppe Bifulco

**Affiliations:** ^1^Department of Neurosciences, Reproductive Sciences and Dentistry, School of Medicine, University of Naples Federico II, Via Sergio Pansini, Naples, Italy; ^2^Department of Public Health, University of Naples Federico II, Via Sergio Pansini, Naples, Italy

## Abstract

**Objective:**

Evaluate the effects of carboxy-methyl-beta-glucan on cervical epithelialization and on the vaginal microbiota in patients with HPV infection or low-grade cervical preneoplastic lesion (CIN 1).

**Materials and Methods:**

Seven-hundred eighty-four women with positive HPV tests or diagnosed with CIN 1 were enrolled in a retrospective case-control study. All the recruited women performed, at baseline and after 6 months, Pap test, HPV test, evaluation of vaginal health according to the Amsel criteria, colposcopy, and punch biopsy. The study population was then divided into 2 groups in relation to the therapy performed during the follow-up period. Group A performed treatment with vaginal gel based on carboxy-methyl-beta-glucan (1 application/day for 20 days per month for 3 months). Group B was the control group.

**Results:**

The patients of group A had a significant improvement in the ectopia pattern and a greater number of cases with metaplasia in the maturation phase with a significant increase in Lugol uptake. In the experimental group, a significant improvement in the pH indices, a negative Swift test and a resolution of the leucorrhoea were observed. A negative result of the 37.1% Pap test and the 39.9% HPV test (vs. 15.2% and 16.5%, respectively) were demonstrated in the treatment group with respect to the control group. A negativization of the colposcopic pictures was observed with a reduction in the amount of CIN 1 found higher in the treatment group.

**Conclusions:**

Vaginal therapy based on carboxy-methyl-beta-glucan has been able to improve overall vaginal health; this effect seemed to positively impact the risk of persistence and progression of CIN.

## 1. Introduction

HPV (human papillomavirus) infection is transmitted mainly by sexual contact, and the cervix is the organ most sensitive to the oncogenic action of papillomavirus. The cervix is covered by two epithelia: the esocervical one and the paved type and the endocervical one and the cylindrical type. This transition zone represents, from a biological point of view, an area of instability because it allows easier access to the basal site of the target reserve cells of oncogenic agents such as HPV [1].

The HPV infection is very common especially in young women (highest incidence peak between 20 and 30 years). There is a percentage of women at risk who are positive for the infection but in whom the virus did not cause cervical precancerous lesions (CIN). When the virus integrates into the cells of the cervical epithelium, it determines a process of cellular transformation and gives rise to the CIN lesions [2, 3].

Recent data show a significant correlation between immune status and persistence of the virus. The vaginal microbiota plays an important role in modulating the immune system of the female genital tract [4]. *Lactobacillus crispatus*, *Lactobacillus gasseri*, *Lactobacillus iners*, and *Lactobacillus jensenii* appear to dominate the vagina of most healthy women. The composition of vaginal microbiota is influenced by numerous factors: ethnicity, cyclical secretion of oestrogen and progesterone throughout the menstrual cycle, menstruation or menopausal state, the widespread use of synthetic hormones for contraceptive purposes, sexual intercourse, hygiene practices, and infection [5].

There is much evidence to correlate the persistence of HPV with the altered presence of vaginal lactobacilli and an altered microbiota. A balanced situation of the vaginal microbiota ensures a better response against HPV [6–9]. Even a well-epithelized cervix, with a healthy epithelium, with a mature and small extension of transformation zone area, offers an environment unfavorable to infection and persistency of the virus.

Current thinking suggests that the development of papillomavirus-associated disease requires the infection not just of an epithelial basal cell but more specifically an epithelial tissue stem cell at a pluristratified cutaneous or mucosal site. At the cervix, the transformation zone is maintained by a specialized type of cell known as the reserve cell. The cervical neoplasia develops primarily at the squamocolumnar junction because these cells fail to properly regulate viral gene expression, leading to a nonproductive or abortive infection rather than a productive infection. The idea that papillomaviruses generally reside in an epithelial stem cell following infection is compatible with our understanding of latency and reactivation from latency. During latency, the virus can be undetectable to the common diagnostic test [1, 10].

Maintaining adequate or improving the state of vaginal health and acting positively on the cervical epithelium and the vaginal microbiota could be a new strategy to prevent both the acquisition and persistence of HPV infection and the progression of CIN lesions.

### 1.1. Purpose of the Study

This study is aimed at evaluating the effects of local therapy with vaginal gel based on carboxy-methyl-beta-glucan on cervical epithelialization and the vaginal microbiota in patients with HPV infection or low-grade cervical preneoplastic lesion.

## 2. Materials and Methods

Seven-hundred eighty-four women with positive HPV tests or diagnosed with CIN 1, referred to the Colposcopy and Cervical Pathology Center of our Department, were enrolled in a retrospective case-control study.

All patients meeting the following enrollment criteria were selected:

- Age between 18 and 60 years
- Caucasian origin
- Positivity to HPV testing and/or positivity to CIN 1 at punch biopsy
- Absence of contraindications to the proposed therapies
- Negative medical history for any type of pharmacological treatment that acts on the elements in the current or recent study (<3 months)
- Negative medical history due to systemic pathology which can influence virus natural history, in particular immune disorders, and diabetes

The exclusion criteria were as follows:

- Age under 18 or over 60 years
- Positivity to biopsy for CIN 2+ lesion
- Suspected or diagnosed hypersensitivity/allergy to the components of the vaginal gel
- A positive medical history for each type of treatment that acts on the current or recent study outcomes (<3 months)
- States of specific deficiency of the immune system

At the time of recruitment, each person was informed about the aims and methods of the study with the fulfillment of a written informed consent. The study was approved by the ethical committee of the Federico II University (*number protocol*: *260/18*).

The data of the patients in the study were extracted from the medical records of the patients who joined the center starting from January 2013.

For all the participants, the previous clinical history, age, specific risk factors, and previous cytology report according to the patient record were evaluated.

All the recruited women have undergone complete clinical examination and subsequent follow-up after 6 months; in particular, the women who performed gynecological and physical examination, Paptest and/or HPV test, and evaluation of vaginal health according to the Amsel criteria (presence of leucorrhoea, vaginal pH > 4.5, positivity of the Swift test, and presence of clue cells).

A targeted punch biopsy during colposcopy was obtained in case of the presence of ZTA (abnormal transformation zone).

The study population was then divided into 2 groups concerning the therapy performed during the follow-up period:

- Group A: 392 women with positive HPV test or CIN 1 diagnosis who performed the treatment with vaginal gel-based carboxy-methyl-beta-glucan (Colpofix®: 1 application/day for 20 days per month for 3 months);
- Group B: 392 untreated women, as the control group

In Group A, the aspects of compliance, treatment security, and the onset of side effects or allergic sensitization were also evaluated.

### 2.1. Statistical Analysis

The number of the sample under examination was calculated starting from the assumption that, as reported in the literature [11], about 60% of low-grade cervical lesions regress spontaneously without any therapy over 12-24 months, and this value is also higher in case of positive HPV test only. Assuming a significant increase of these regressions of at least 10% in the course of adjuvant therapy, setting the calculation with an alpha-error at 5% (CI. 95%) and a 1 − *β* = 80% the sample size must be of at least 356 patients per group. Considering a 10% dropout (for lack of data completeness), it was decided to increase this value to 392 patients.

SPSS software (version 20.0) was used for statistical analysis. The significance was set at a value of *p* < 0.05.

The demographic and clinical data of the two groups were compared with the Student *t*-test for data with parametric distribution (age, weight) and with the *χ*^2^ test for ordinal variables (Pap test result, vaginal swab result, and colposcopic report). The differences in the number of CIN lesions and HPV test positivity between the two groups were evaluated with the *χ*^2^ test. The measurement of the OR (odds ratio) was used to assess the risk of persistence of infection or lesions.

## 3. Results

At the end of the data analysis, 725 patients were found to meet the inclusion criteria and completed the follow-up as required by the protocol: 358 belonging to group A, 367 belonging to group B. The demographic and anamnestic variables of the subjects under study are reported in Table 1. The two groups were comparable to the aforementioned variables (*p* = NS).

**Figure 1:**
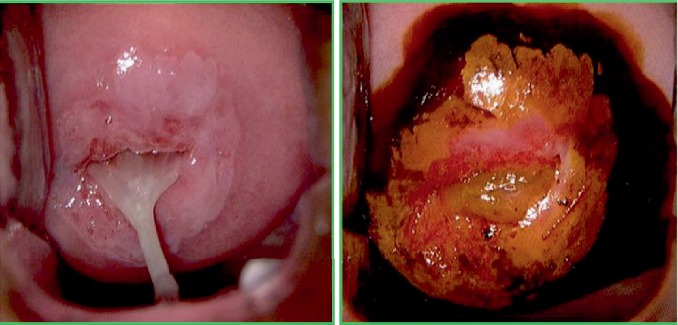
Abnormal transformation zone (ATZ)—CIN 1 before treatment with carboxy-methyl-beta-glucan.

**Figure 2:**
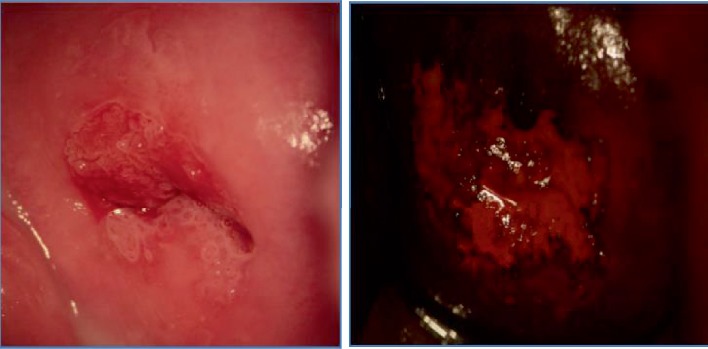
Normal transformation zone (NTZ) after treatment with carboxy-methyl-beta-glucan.

**Figure 3:**
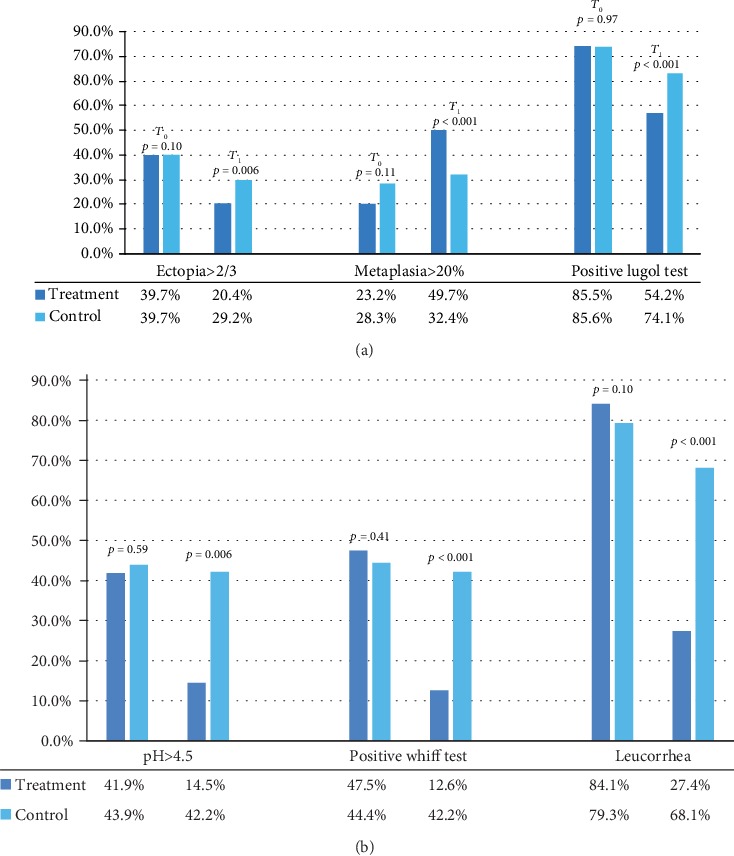
(a) Epithelialization and vaginal health indexes in the two study groups. (b) Epithelialization and vaginal health indexes in the two study groups.

As regards the vaginal and tissue health outcomes, it was observed that the patients who performed the therapy, concerning the control group, had a significant improvement in the ectopia pattern (Figures 1 and 2): a greater extension (> 20%) of metaplasia and a greater number of cases with metaplasia in the maturation phase with a significant increase in Lugol uptake (Table 2 and Figure 3(a)).

In the experimental group, a significant improvement in the pH indices, a negative Swift test, and a resolution of the leucorrhoea were observed (Table 2 and Figure 3(b)).

Table 3 shows the cytohistological, molecular, and colposcopic data of the patients in the study. A negative result of the 37.1% Pap test and the 39.9% HPV test (15.2% and 16.5%, respectively) were demonstrated in the treatment group to the control group. Consistent with these data, a negativization of the colposcopic pictures is observed with a reduction in the amount of CIN 1 found higher in the treatment group. Overall a CIN 1 lesion regression is observed in the total population in 6 months of about 20% (from 63.2% to 48.0%).

**Figure 4:**
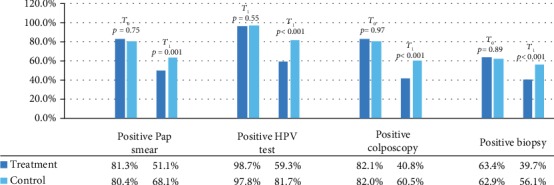
Cytological, molecular, colposcopic, and histological reports in the two study groups.

No adverse effects were reported by patients in therapy (Figure 4).

The analysis of risk factors for the persistence of infection or lesions (Table 4) showed that, among the lifestyle risk factors, cigarette smoking (more than 10 sig/die) leads to an increase in the risk of persistence of the lesion about 2 times. The presence of ectopia, a vaginal pH greater than 4.5, and a positive Swift test entail a doubled risk of the persisting lesion, and the presence of leucorrhoea carries a risk of 3.5 times greater than the persistence of lesion. Performing the treatment in reverse halves this risk.

## 4. Discussion

Many factors have been reported regarding the risk of persistence of HPV infection and the progression of a CIN lesion. In recent years, many studies have shown that the stability and composition of the vaginal microbiome can influence viral clearance and probably also the progression of cervical preneoplastic lesions [1, 4–9].

The treatment used is based on carboxy-methyl-beta-glucan, hydrophilic polymers capable of forming on the vaginal mucosa a mucoadhesive film that protects from external microbial agents and assists in maintaining and controlling the physiological conditions of the processing areas of the cervicovaginal mucosa by hydration. Carboxy-methyl-beta-glucan contributes to the maintenance and/or restoration of the vaginal microbiota through a prebiotic effect.

Our data have shown how in vivo carboxy-methyl-beta-glucan in polycarbophil vaginal therapy can improve cervical epithelialization. Indeed, an improvement in the ectopia pattern was observed with a reduction in severe ectropion (>2/3) in the therapy group compared to the control group, with a corresponding increase in the rate and extension of metaplasia and an improvement in the uptake of the Lugol test with a reduction in the proportion of patients with a noncaptive test.

The observed metaplasia and Lugol's test uptake were expressions of the repair process of ectopia with the conversion of a delicate tissue such as the cylindrical one into tissue more resistant to vaginal insults such as the multilayered squamous tissue.

This repair probably involves a reduction in the rates of tissue inflammation and predisposition to the development of infection. Indeed, the therapy has been shown to improve the vaginal health of patients who presented clinical signs of bacterial or fungal vulvovaginitis at the first visit. The therapy induced a resolution of the leucorrhoea, a normalization of the vaginal pH, and a negativization of the Swift test in a more significant percentage of cases compared to the group not in therapy.

Our data demonstrated a regression rate at 6 months from the histological diagnosis of CIN 1 significantly higher in the therapy group (23.7%; *n* = 85/358) than in the control group (6.8%; *n* = 25/367).

The literature reported a rate of spontaneous CIN 1 lesion remission of about 60% over a 12-24-month period [11]. Our data agree with the data of Scardamaglia et al. [12] and Stentella et al. [13] who demonstrated a significant regression of CIN 1 lesion in patients treated with carboxymethyl beta-glucan.

It is likely that the improvement observed in the study population was attributable to the therapy performed as the short follow-up interval (6 months). The effect probably depended on the ability of the medical device to promote greater stability of the vaginal environment.

The presence of bacterial vaginosis (VB) has been associated with a delay in HPV clearance in patients with CIN, suggesting that the presence of a poor lactobacilli microbiota (community state type-IV-CST IV) may play a role in this persistence. It was also shown that greater diversity of the vaginal microbiota (CST-IV) is associated with greater severity of CIN lesions [14].

The presence of lactobacilli-producing H_2_O_2_ seems protective in preventing the progression of dysplasia and ultimately the carcinogenic process. A greater prevalence of L. jensenii and L. coleohominis, both producers of H_2_O_2_, has been demonstrated in women with LSIL compared to HSIL. Furthermore, regardless of lactic acid concentrations, Lactobacilli spp. is capable of being cytotoxic when cultured in vitro against cervical cancer cells, but not in normal cells, highlighting an even more complex interaction between cervical cells, the microbiota, and the vaginal environment [6].

This study has shown that correcting the vaginal microbiota and favoring cervical epithelialization with a specific therapy favors CIN 1 lesion regression.

Our data confirmed that the presence of leucorrhoea, a positive Swift test, and a basic vaginal pH correlate with an at least doubled risk of persistence of CIN lesion at the cervical level.

Active and passive cigarette smoking is one of the major risk factors in the development of CIN in the presence of HPV infection [15]. Min et al. have recently shown that smokers have an increased risk of both CIN 1 (OR = 1.81; 95% CI, 1.26–2.60) and CIN 2/3 (OR = 1.77; 95% CI, 1.10-2.86) [16].

Alcohol consumption is known as a potential risk factor in acquiring HPV infection, although the data on the risk of persistence of lesions, the presence of cervical cancer, and alcohol consumption are few and controversial [17–19]. In our study, we showed an increased risk of persistent lesions in smokers but not in the presence of alcohol consumption.

The high number of patients, the execution of diagnostic tests (always performed by the same operators), and the short follow-up times (6 months) represented the strengths of this study.

The choice of enrolling only patients with reassessment time at 6 months from the first visit has allowed to minimize the variations of the vaginal environment induced by other environmental factors and to exclude that the observed improvement of colposcopic pictures and histological findings may be linked to the natural history of HPV infection. The choice of enrolling only Caucasian patients in the study erases the impact of ethnicity on the risk of CIN and cervical microbiota differences in other populations. Indeed, it has been demonstrated that the overall HPV prevalence rate of migrant women is four times higher than the overall prevalence observed among Italian women [20].

The limitations of the study were its retrospective nature and the lack of randomization with a placebo group.

In conclusion, vaginal therapy based on carboxy-methyl-beta-glucan turned out to improve overall vaginal health with a positive impact on the risk of persistence and progression of low-grade cervical lesions of the uterine cervix.

## Figures and Tables

**Table 1:** The demographic and anamnestic variables of the subjects under study.

	Groupp A (*N* = 358)	Group B (*N* = 367)	*p* value
Age (year)^§^	34.02 ± 6.5	34.2 ± 6.2	0.71
Parity (no.)^∗^	1 (0-3)	1 (0-3)	0.52
Educational level^∗^	2 (1-3)	2 (1-3)	0.64
Smoke°			
≤10 sig/die	190 (53.1)	187 (52.2)	0.81
>10 sig/die	168 (46.9)	171 (47.8)	
Alchohol habits°			
Weekly	301 (84.1)	318 (86.6)	0.33
Daily	57 (15.9)	49 (13.4)	
Sexual partners°			
≤4	94 (26.3)	112 (30.5)	0.20
>4	264 (73.7)	255 (69.5)	

**°** *χ* square, ^§^*t*-test, ^∗^Mann–Whitney.

**Table 2:** Epithelialization and vaginal health indexes in the two study groups.

	Time 0 months		*p* value	Time 6 months		*p* value	Difference % group A	Difference % group B
	Group A (*N* = 358)	Group B (*N* = 367)		Group A (*N* = 358)	Group B (*N* = 367)			
Ectopia			0.10			0.006		
≤2/3	216 (60.3)	244 (66.5)		285 (79.6)	260 (70.8)			
>2/3	142 (39.7)	123 (39.7)		73 (20.4)	107 (29.2)		-48.6%	-13%
Metaplasia			0.11			<0.001		
≤20%	275 (76.8)	263 (71.7)		180 (50.3)	248 (67.6)			
>20%	83 (23.2)	104 (28.3)		178 (49.7)	119 (32.4)		114.5%	14.4%
Lugol test			0.97			<0.001		
Negative	52 (14.5)	53 (14.4)		164 (45.8)	95 (25.9)			
Positive	306 (85.5)	314 (85.6)		195 (54.2)	272 (74.1)		-36.3%	-13.4
pH			0.59			<0.001		
≤4.5	208 (58.1)	206 (56.1)		306 (85.5)	212 (57.8)			
>4.5	150 (41.9)	161 (43.9)		52 (14.5)	155 (42.2)		-65.3%	-3.7%
Swift test			0.41			<0.001		
Negative	188 (52.5)	204 (55.6)		313 (87.4)	212 (57.8)			
Positive	170 (47.5)	163 (44.4)		45 (12.6)	155 (42.2)		-73.5%	-4.9%
Leucorrhoea			0.10			<0.001		
Absent	57 (15.9)	76 (20.7)		260 (72.6)	117 (31.9)			
Present	301 (84.1)	291 (79.3)		98 (27.4)	250 (68.1)		-67.4%	-14.1%

**Table 3:** Cytological, molecular, colposcopic, and histological reports in the two study groups.

	Time 0 months		*p* value	Time 6 months		*p* value	Difference % group A	Difference % group B
	Group A (*N* = 358)	Group B (*N* = 367)		Group A (*N* = 358)	Group B (*N* = 367)			
Pap smear			0.75			<0.001		
Negative	67 (18.7)	72 (19.6)		175 (48.9)	117 (31.9)			
Positive	291 (81.3)	295 (80.4)		183 (51.1)	250 (68.1)		-37.1%	-15.2%
HPV test			0.55			<0.001		
Negative	2 (1.3)	4 (2.2)		61 (40.7)	33 (18.3)			
Positive	148 (98.7)	176 (97.8)		89 (59.3)	147 (81.7)		-39.9%	-16.5%
Colposcopy								
Negative	64 (17.9)	66 (18.0)	0.97	212 (59.2)	145 (39.5)	<0.001		
Positive	294 (82.1)	301 (82.0)		146 (40.8)	222 (60.5)		-50.3%	-26.2%
Biopsy								
Negative	131 (36.6)	136 (37.1)	0.89	216 (60.3)	161 (43.9)	<0.001		
Positive	227 (63.4)	231 (62.9)		142 (39.7)	206 (56.1)		-37.4%	-10.8%

**Table 4:** Relative risk of persistence of injury in relation to the variables under study.

	Case	Total	RR (IC 95%)
Smoke habits > 10 cig	216	384	2.04 (1.51-2.74)
Daily alcohol use	46	106	0.81 (0.53-1.21)
Partner > 4	255	519	1.17 (0.85-1.62)
Ectopia > 2/3	117	180	2.52 (1.78-3.58)
Metaplasia > 20%	145	297	1.06 (0.78-1.42)
Lugol test positive	305	466	9.52 (6.51-13.90)
pH > 4.5	126	207	2.07 (1.49-2.88)
Wift test positive	122	200	2.07 (1.48-2.89)
Presence of leucorrhoea	222	348	3.51 (2.58-4.76)
Treatment	142	358	0.51 (0.38-0.69)

## Data Availability

The data used to support the findings of this study are available from the corresponding author upon request.
